# A computational model for GPCR-ligand interaction prediction

**DOI:** 10.1515/jib-2019-0084

**Published:** 2020-12-29

**Authors:** Shiva Karimi, Maryam Ahmadi, Farjam Goudarzi, Reza Ferdousi

**Affiliations:** Health Information Management Department, Paramedical School, Kermanshah University of Medical Sciences, Kermanshah, Iran; Department of Health Information Management, School of Management and Medical Information Sciences, Iran University of Medical Sciences, Tehran, Iran; Regenerative Medicine Research Center, Kermanshah University of Medical Sciences, Kermanshah, Iran; Department of Health Information Technology, School of Management and Medical Informatics, Tabriz University of Medical Sciences, Tabriz, Iran

**Keywords:** drug targeting, GPCR, interaction, ligand, machine learning

## Abstract

G protein-coupled receptors (GPCRs) play an essential role in critical human activities, and they are considered targets for a wide range of drugs. Accordingly, based on these crucial roles, GPCRs are mainly considered and focused on pharmaceutical research. Hence, there are a lot of investigations on GPCRs. Experimental laboratory research is very costly in terms of time and expenses, and accordingly, there is a marked tendency to use computational methods as an alternative method. In this study, a prediction model based on machine learning (ML) approaches was developed to predict GPCRs and ligand interactions. Decision tree (DT), random forest (RF), multilayer perceptron (MLP), support vector machine (SVM), and Naive Bayes (NB) were the algorithms that were investigated in this study. After several optimization steps, receiver operating characteristic (ROC) for DT, RF, MLP, SVM, and NB algorithm were 95.2, 98.1, 96.3, 95.5, and 97.3, respectively. Accordingly final model was made base on the RF algorithm. The current computational study compared with others focused on specific and important types of proteins (GPCR) interaction and employed/examined different types of sequence-based features to obtain more accurate results. Drug science researchers could widely use the developed prediction model in this study. The developed predictor was applied over 16,132 GPCR-ligand pairs and about 6778 potential interactions predicted.

## 1 Introduction

Peptides and proteins with different structures and various biological impress play an essential role in the human body’s main activities (e.g., hormones, neurotransmitters, modulators) and diagnoses/therapy of the disease [1], [2], [3]. The main part of target proteins and nearly more than half of drug targets are enzymes and G protein-coupled receptors (GPCRs). According to the literature, GPCRs are an interesting group of druggable proteins that are vastly considered drug targets (almost 26.8% of drug targets are GPCRs) [2], [4], [5]. GPCRs are classified in five distinct groups (i.e., glutamate [G], rhodopsin-like [R], adhesion [A], frizzled/taste [F] and secretin [S]) [6]. Moreover, GPCRs are the biggest receptor family among all the cell surface receptors [7]. A significant part of GPCR-targeted drug development is related to an interaction between GPCRs and other molecules (e.g., natural ligands, synthetic agonists, and other proteins) [4].

Selected/designed ligands based on their functions could be used as new therapies and could lead to hope for drug discovery [8]. Hence, product development has various troubles and problems that led to a significant failure rate in the biopharma industry. Therefore, there is a long timeline to investigate and develop new pharmaceutical products (nearly 8–10 years) and need vast R&D investments. Attrition rates as high as 90% exist for the clinical trial stages after the research stages that dramatically increase drug development costs [9]. The following table (Table 1) shows the estimated costs for drug development over the past four decades [9].

**Table 1:** The estimated cost for drug development over the past four decades.

Year	1980	1990	2014
Estimated drug development cost	413 million $	1.04 billion $	2.59 billion $

Recently, with the advancement in computational methods and the popularity of computational methods in protein-ligand interactions, considerable parts of laboratory experiments that are expensive in terms of time and costs were omitted [10]. Nowadays, GPCR studies led to new findings that can result in novel drug discovery.

The impact of accurate and adequate information on the utilization of machine learning (ML) approaches is very high. Protein sequences are one of the most reliable information resources for drugs and targets that many functional features could be extracted from protein sequences [11]. The proteins’ functional and biological activities are influenced by a specific section of their sequences called domains. Domains in sequences are known as segments with known functions [12], [13], [14], [15], [16]. Conserved domain models are based on multiple sequence alignments of related proteins that reveal sequence regions containing similar amino acids patterns [17].

In this study, specific quantitative data were extracted based on sequences (e.g., amplitudes, clustering, and various amino acid combinations). Various ML algorithms with a different mixture of data sets were examined and evaluated. The models were compared according to their performance, and the model with the highest performance employed to predict potential interacting pairs. Choosing the right data set, performing step-by-step modeling, and evaluating them helped us achieve better results and reliable model [17], [18], [19].

## 2 Methods

### 2.1 Data acquisition

Tuples construction: GPCRs list retrieved based on keyword <Family: “G Protein-Coupled Receptor” AND Reviewed: “Yes”> in UniProt protein database available at www.uniprot.org [20]. Table S1 in the supplementary file shows the list of acquired GPCRs. This list was matched with core GPCRs interactions accessible at the database of interacting protein (DIP) [21]. To do this, all interaction pairs in DIP (Table S2) that at least one of them was GPCR (based on Table S1) were extracted, and the rest of them eliminated. Finally, 224 interactions were extracted (Table S3). These pairs were considered as pairs that there is DIP confirmed evidence for the interaction between them. About 116 receptors and 141 ligands were in the list of selected interactions (Table S4 and S5). From obtained receptors and ligands, 16356 possible GPCR-ligand pairs were constructed (Table S6). ML algorithms need negative tuples (pairs that have no theoretical/practical evidence for interaction between them). Therefore, to make negative examples for the ML algorithms’ training, a random selection method was employed to make a list of negative tuples for the training of the ML algorithms (Table S7).

### 2.2 Feature extraction and combination

The proteins’ sequences are the basis for feature extraction in many studies [22], [23], [24], [25], [26]. GPCRs and ligands sequences were extracted from UniProt. The sequences are strings made of 20 amino acids. The conserved domains of GPCRs and ligands were the information sources employed in this work to extract features about GPCRs and ligands. NCBI conserved domain database (CDD) [27] was used to extract domains. In this regard, domains with E-VALUE = 0.01 were extracted from CDD. Two types of domains including, specific and non-specific domains in various confidence levels, were extracted. Moreover, two domain model scopes, including super-families and multi-domains, were extracted for each sequence. Definitions below are adopted from the NCBI database webpage [17]:

Specific domain: the top-ranking hit (compared to other hits in overlapping intervals) that meets or exceeds a domain-specific E-value threshold (details and illustration). It represents very high confidence that the query sequence belongs to the same protein family as the sequences used to create the domain model, and therefore a high confidence level for the inferred function of the protein query sequence.

Non-Specific domain: If a specific hit was not found on a query protein sequence, but the protein has an otherwise statistically significant hit (E-value cutoff of 0.01) to any domain model in CDD, the domain model is regarded as a non-specific hit.

Super-family is the domain cluster to which the specific and/or non-specific hits belong. Super-family is a set of conserved domain models that generate overlapping annotation on the same protein sequences and are assumed to represent evolutionarily related domains.

Multi-domains: Multi-domains are domain models that were computationally detected and are likely to contain multiple single domains.

**Figure 1:**
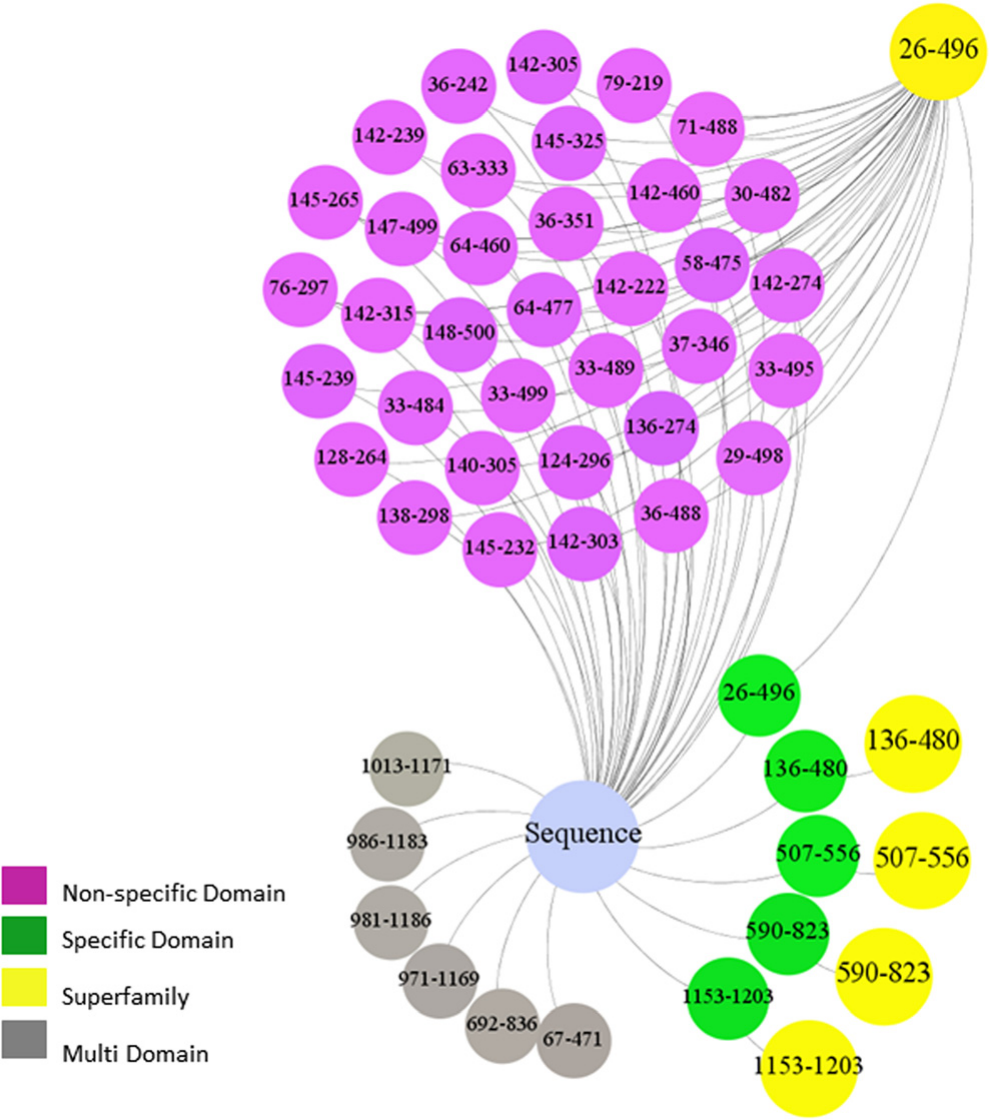
Extracted domains of guanine nucleotide-binding protein (UniProt id: P10823).

For every sequence, four groups of features, including specific domains, non-specific domains, multi-domains, and super-family, were extracted (see Tables S8–S11). Figure 1 shows extracted features based on conserved domains for guanine nucleotide-binding protein (UniProt id: P10823).

Finally, to determine training capability for all four groups of features, different datasets with all possible compositions of the feature groups were constructed. The algorithms’ performance with different data sets were evaluated based on the area under the curve (AUC).

### 2.3 Parameter setting

Optimization parameters of the algorithms with different values were examined, and then the best parameters for optimal performance were selected. In this study, for the decision tree (DT) algorithm, K, and D parameters, for the random forest (RF) algorithm I, K, and S parameters, for multi-layered perceptron algorithms number of nodes, hidden layer, learning rate, and momentum parameters, and for support vector machine (SVM) C and gamma parameters were optimized.

### 2.4 Feature selection

Feature selection (FS) approaches were used to improve the predictive capability of employed models. Relief, information gain, and SVM methods were used for FS. First, all the features ranked based on FS algorithms, and then the feature with the lowest importance (based on FS algorithms) was removed. After each feature elimination, the performance of the prediction models was measured. Elimination of the features and performance measuring was continued until the best set of features was determined.

### 2.5 Prediction model construction

After determining the best feature group and determining the best parameter setting for prediction models based on their performance over training examples, the best prediction model was selected and constructed. Five common ML algorithms, including SVM, multilayer perceptron (MLP), Naive Bayes (NB), RF, and DT were evaluated based on their performance. All five algorithms with different feature sets were examined and compared to determine the best predictor. Algorithms performance assessed based on the standard assessment criteria such as accuracy and receiver operating characteristic (ROC).

### 2.6 Measurements and the performance evaluation of models

In this section, details of the performance evaluation criteria were explained. A 10-fold cross-validation method was used to evaluate the performance of the algorithms. Also, 70% of the data were used as a training set, and the remaining 30% were used as a test set. Recruited performance evaluation measures in this study are as follow:

True Positive (TP): interacting receptor-ligand pairs that correctly identified as interacting pairs.

False Positive (FP): non-interacting receptor-ligand pairs that incorrectly identified as interacting pairs.

True Negative (TN): non-interacting receptor-ligand pairs that correctly identified as non-interacting pairs.

False Negative (FN): interacting receptor-ligand pairs that incorrectly identified as non-interacting pairs.

Accuracy (ACC): The percentage of interactive and non-interactive pairs that are predicted correctly.

$$\text{ACC}=\frac{\text{TP}+\text{TN}}{\text{TP}+\text{FP}+\text{TN}+\text{TP}}$$

Sensitivity (SN): The percentage of interactive pairs that are properly predicted.

$$\text{SN}=\frac{\text{TP}}{\text{TP}+\text{FP}}$$

Specificity: The percentage of non-interactive pairs that are predicted correctly.

$$\text{SP}=\frac{\text{TN}}{\text{TN}+\text{FP}}$$

The operating characteristic curve area (AU-ROC) was also calculated to measure the performance of the models. AU-ROC is often used to report the performance of binary predictors [28].

### 2.7 Prediction of potential GPCR-ligand interactions

In this step, the constructed model based on the previous section was used to predict the potential interaction of GPCR-ligand pairs. The model was applied on all GPCR-ligand pairs with unknown interaction label to predict potential GPCR-ligand pairs.

### 2.8 Software

In this study, Weka (version 3.8.0) was used for data mining purposes, and Gephi (version 0.9.1) was used for graph plotting.

## 3 Results & discussion

### 3.1 Data acquisition result

**Figure 2:**
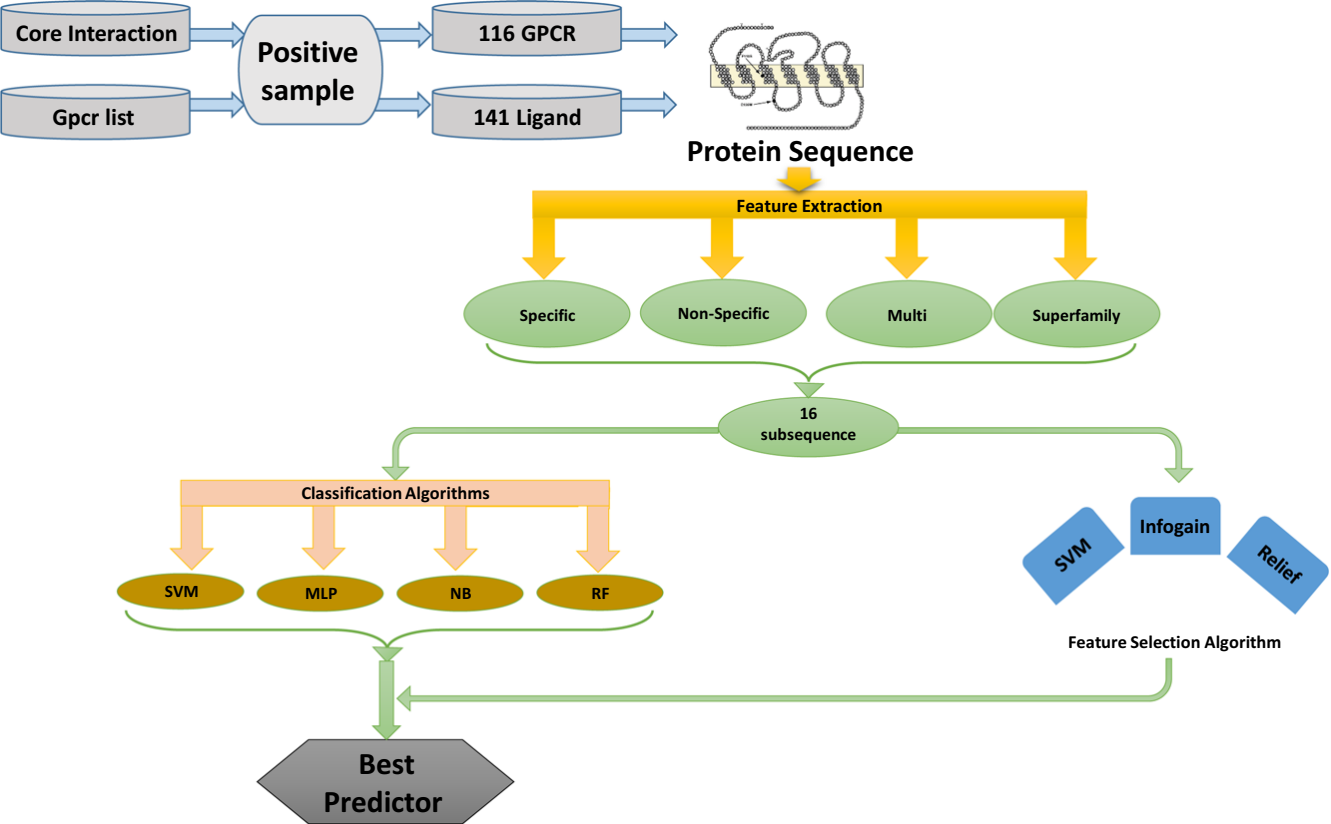
Overall illustration of the proposed approach for GPCR-ligand interacting pairs.

This study utilized the capability of ML approaches to predict GPCR-ligand interaction. Known (experimentally confirmed) GPCR-Ligand interactions used to train the algorithms (see Tables S1–S7 in supplementary file). The overall process of this study is described in Figure 2.

### 3.2 Feature extraction and combination

Based on the mentioned feature extraction methodology mentioned earlier, five groups of the features (A total of 3118 features) were extracted. Table 2 shows the extracted feature groups in detail.

**Table 2:** Extracted feature groups for GPCRs and ligands.

Features	GPCRs	Ligands
k-mer=> *k* = 1, *k* = 2	20, 400	20, 400
Specific domain	44	192
Non-specific domain	99	1416
Super-family	34	150
Multi-domain	39	304

The performance of the five ML algorithms with varying groups of features was assessed based on AU-ROC (Table 3).

**Table 3:** The algorithms performance evaluation based on AU-ROC with different features set (bold numbers indicates the best performance in each column).

Algorithm	SVM			RF	NB	DT	MLP
	Linear	Quadratic	RBF				
Specific D	85.5	86.2	78.6	92.8	88.6	89.6	87.7
Non-specific D	84.8	85	80.4	92.3	85.8	86.7	58.3
Superfamily	94	**95.1**	95.3	97	**96.6**	**93.2**	**95.3**
Multi D	**94.4**	93.8	**95.5**	95.9	84.1	92.6	95
One and two mer	93.5	93.1	95.3	**97.1**	94.3	89.7	94.8
**MAX AU-ROC**	**94.4**	**95.1**	**95.5**	**97.1**	**96.6**	**93.2**	**95.3**

Among all the examined algorithms, RF algorithm with AU-ROC = 97.1 had the best performance. Moreover, among different feature groups, features based on k-mer method and super-families presented better predictive capabilities (Table 3). All possible combinations of the five feature groups were also examined to utilize various feature groups’ predictive capabilities. Table 4 shows the performance of all ML algorithms with all possible feature groups. More details about the algorithm’s evaluation are available in the Supplementary Table S12.

**Table 4:** Algorithms performance comparison with different feature groups (bold numbers indicates the best performance in each column).

Feature groups	SVM			RF	NB	DT	MLP
	Lin	Quad	RBF				
Specific D – Non-specific D	93.5	94.2	95.3	97.5	92.7	93	55
Specific D – Superfamily	92.6	**95.1**	95.3	97	**96.6**	93	70.6
Specific D – one- two Mer	93.1	93.1	95.3	97.3	94.4	89.2	94.2
Specific D – Multi D	93.1	94.9	**95.5**	97.4	95.3	93.1	66
Non-specific D – Superfamily	93.1	94.4	**95.5**	**97.7**	93.7	93.1	58
Non-specific D – one- two Mer	92.4	93.1	**95.5**	97	94.3	86.2	83.9
Non-specific D – Multi D	93.5	94	95.3	97.1	92.1	91.9	71.2
Superfamily – one- two Mer	93.5	93.1	95.3	97.6	94.4	89.6	**94.6**
Superfamily – Multi D	93.5	94.6	**95.5**	97.4	96.2	92.4	72.6
One- two Mer – Multi D	93.1	93.5	95.3	97.2	94.4	89.2	94.3
Specific D – Non-Specific D – Superfamily	93.5	94.6	95.3	97.6	94.1	92.8	54.2
Specific D – Non-Specific D – one- two Mer	92.6	92.6	**95.5**	97.1	94.3	89.6	92.5
Specific D – Non-Specific D – Multi D	**93.8**	94.4	95.3	97.6	93.2	91.8	66.6
Specific D – Superfamily – one- two Mer	93.1	93.1	95.3	97	94.4	89.6	94.3
Specific D – Superfamily – Multi D	93.3	94.9	**95.5**	97.5	95.4	92.7	61.6
Specific D – one- two Mer – Multi D	92.4	93.8	95.3	97.2	94.4	89.2	93.8
Non-specific D – Superfamily – one- two Mer	92.4	93.3	**95.5**	97.3	94.3	89.6	74.2
Non-specific D – Superfamily – Multi D	93.3	94	95.3	97.5	93.7	91.8	67.3
Non-specific D – one- two Mer – Multi D	92.4	93.3	95.3	97.1	93.7	89.6	80.2
Superfamily – one- two Mer – Multi D	93.1	93.5	95.3	97.4	93.7	89.6	94.2
Specific D – Non-specific D – Superfamily – one- two Mer	92.6	92.6	**95.5**	97.4	94.3	89.6	82
Specific D – Non-specific D – Superfamily – Multi D	93.5	94.4	95.3	**97.7**	94.2	91.8	66.9
Specific D – Non-specific D – One- two Mer – Multi D	92.6	94	95.3	96.9	93.7	89.6	84.1
Specific D- Superfamily- one- two Mer- Multi D	93.3	94.4	94.9	96.9	94.5	**94.5**	87.3
Non-specific D – Superfamily – one- two Mer – Multi D	92.6	93.8	95.3	97.3	93.7	89.6	61.6
Specific D – Non-specific D – Superfamily – one- two Mer – Multi D	92.6	94	95.3	96.9	94.5	86.2	85.1
**MAX AU-ROC**	**93.8**	**95.1**	**95.5**	**97.7**	**96.6**	**94.5**	**94.6**

Table 4 shows that, in some experiments, some algorithm’s performance with combinatory features were improved. However, some algorithm’s performance with combinatory features were deteriorated (i.e., linear SVM and MPL). Table 5 provides information about the algorithms’ performance before and after the combination of the feature groups.

**Table 5:** Algorithms’ performance before and after the combination of the feature groups.

	SVM			RF	NB	DT	MLP
	Linear	Quadratic	RBF				
Before feature combination	94.4	95.1	95.5	97.1	96.6	93.2	95.3
After feature combination	93.8↓	95.1	95.5	97.7↑	96.6	94.5↑	94.6↑

At the end of this step, a set of features with best performance was selected for each algorithm (Table 6).

**Table 6:** The selected subsets for each algorithm.

Algorithms	Best subsets
SVM(RFB)	Multi D
RF	Non-specific D – Super-family
NB	Super-family
DT	Specific D – Super-family – one-two Mer – Multi D
MLP	Super-family

### 3.3 Parameter setting

Different parameters and settings for each algorithm were examined to obtain a more accurate model (Table 7). For example, the values for trees, feature, and seed were investigated for the RF algorithm.

**Table 7:** Investigating the result of different values for parameter and settings on algorithm performance (bold numbers indicates the value of parameters best performance in each column).

	SVM (RFB)	RF	NB	DT	MLP	MAX AU-ROC
Gamma = 0.001, **0.01**, 0.1, 1, 10, 100, 1000	*					95.5
*C* = 0.001, **0.01**, 0.1, 1, 10, 100, 1000						
*I* (Number of three) = 10, 100, **1000**		*				97.8
*K* (number of feature) = 1, 2, **3**, … , 10						
*S* (seed) = **1**, 2, 3, 4						
K = kernel density estimator (mode)			*			97
**D** = supervised discretization (mode)						
Confidence rate = 0.1, **0.2**, 0.3 , … , 1.0				*		95.6
Minimum number of instance = 1, 2, 3, 4, 5, **6**, … , 15						
Layer = 1, 2, **3**					*	96.3
Node = 1, 2, 3, … , **12**, … , 30						
Learning rate = 0.1, 0.2, **0.3** , … , 1.0						
Momentum = 0.1, **0.2**, 0.3 , … , 1.0						

*Indicates which parameters algorithms are related to which algorithms.

#### 3.3.1 SVM (RBF)

For the RBF algorithm, the parameters including Gamma and C were examined (on multi-domain subset), and the default values of these two parameters (C = 1, Gamma = 0.01) were replaced with numbers 0.001, 0.01, 0.1, 1, 10, 100, and 1000. The best performance was AU-ROC = 95.5. The SVM(RBF) algorithm had best performance with default values (i.e.,. C = 1, Gamma = 0.01). for more details, please see Table S13 in supplementary file.

#### 3.3.2 RF

Different numbers for trees, feature, and seed parameters were examined to achieve the best performance for the RF algorithm. For this algorithm, I, K, and S parameters were set as 100, 3, and 1, respectively. With the new setting, the performance of the algorithm was improved (see Table S14).

#### 3.3.3 NB

The performance of the NB algorithm with different settings was investigated. For kernel density estimator (K), normal distribution for numeric attributes was used, and for the supervised discretization to process (D), numeric attributes were used (see Table S15).

#### 3.3.4 DT

Pruning confidence and minimum number instances were the parameters that were optimized for the DT algorithm. DT performance with PC = 0.2 and MI = 6 was improved from AU-ROC = 94.5–95.6 (see Table S16 in supplementary file).

#### 3.3.5 MLP

Learning rate, momentum, number of hidden layers, and number of nodes are the parameters that were optimized for the MPL algorithm. There was a decrease in AU-ROC value and performance of the MLP algorithm by changing the learning rate and momentum. These two parameters with default values had better performance for the algorithm. However, by changing the number of nodes and layers, the performance was improved. A three layered network with 12 nodes led to an AU-ROC = 96.3 (see Table S17 in supplementary file).

### 3.4 Feature selection

For each algorithm’s best feature set was determined, and then three FS methods (i.e., Info Gain, relief, and SVM) were performed in the last step of the optimization process. At first, features ranked based on FS, and 10 features with the lowest impact were eliminated, and the algorithms’ performance was measured. The following table (Table 9) summarizes the results of performing FS. More details are available in Table S18 in the supplementary file.

**Table 8:** Summary of algorithm parameters optimization impact on the overall performance of the algorithms.

	SVM (RFB)	RF	NB	DT	MLP
Before parameters optimization	95.5	97.7	96.6	94.5	95.3
After parameters optimization	95.5	97.8↑	96.6	95.6↑	96.3↑

**Table 9:** Comparison of the performance of algorithms after feature selection.

	Info gain		Relief		SVM	
	AU-ROC	NUM	AU-ROC	NUM	AU-ROC	NUM
RBF (Multi domain)	95.5	104	95.5	84	95.5	5
MLP (Superfamily)	96.3	185	96.5	25	96.3	185
RF (Specific D – Non-Specific D – Superfamily – Multi D)	97.8	1420	**98.1**	870	97.9	1440
NB (Superfamily)	97.3	45	97	125	96.6	125
DT (Specific D – Superfamily – one- two Mer – Multi D)	95.6	1354	95.6	1204	95.6	1484

The bold number shows the best result of the same parameter.

### 3.5 Prediction model construction and identifying GPCRs-ligands pairs with the potential to interact

RF with AU-ROC = 98.1 was selected as the best prediction model. The final model was constructed based on the RF algorithm with selected features from specific domains, non-specific domains, super-family, and multi-domains (870 features). Finally, a constructed model was applied over 16,132 GPCR-ligand pairs, and 13,295 potential interactions were identified.

## 4 Discussion

Introducing new protein-ligand interaction could be very valuable in developing new drugs and could lead to a better understanding of protein function. Also, it can [29]. The importance of the GPCR proteins family in the pharmaceutical industry and disease treatment is considerably high [30], [31], and GPCR-protein interactions prediction has always been at the center of attention. In this study, we trained five supervised machine-learning-based algorithms with various data sets. This study’s recruited data were various compounds of the amino acids, various protected domains, and different clustering of them. Domains are the essential functional dimensions of proteins sequence that have been found in many studies that recognize their function [32], [33], [34], [35], [36], [37], [38]. Therefore, the domains for GPCR and ligands were extracted from a valid database (i.e., CDD, which collects domains of protein sequences). Super-family is another information source used in this study to train the ML algorithms. Super-families are non-repeat protected domains gathered in a cluster [39].

In this study, different approaches (i.e., combining feature groups, optimization of the algorithm parameters, and FS) were used to increase the accuracy of ML algorithms. Optimization processes in every aspect of the learning phase were led to constructing a reliable prediction model [17]. This step by step optimizations was practical (Tables 4, 7, and 8), and the accuracy of all algorithms was increased (overall AU-ROC > 0.95).

The best performance (AU-ROC = 98.1) was recorded for RF algorithm on the selected feature (i.e., a combination of specific domains, non-specific domains, and super-family and multi-domains). Therefore, the prediction model was constructed based on the RF algorithm with the mentioned feature set. The constructed model applied on 16,132 pairs of GPCR-ligand, and 6778 pairs of potential interaction were identified.

## 5 Conclusion

New technologies make it possible to perform soft computing approaches in biomedicine and pre-laboratory screening, which could be significant in terms of time and cost. For example, suppose the goal is to identify a ligand that could bind to specific proteins. In that case, the interactions could be evaluated computationally before any *in vivo* or *in vitro* experiments.

This study also introduced pairs of potential GPCR-Ligand interactions, which could significantly increase the chances of success in related laboratory researches. Future studies could confirm potential GPCR-ligand interactions that resulted in this study. Hence, these interactions could be recruited in many biomedical investigations (e.g., the transmission of biological messages, creating signaling pathways, and developing new drugs).

The lack of experimental samples for non-interactive pairs was a limitation for this study. Accordingly, having a list of non-interactive pairs could help in the construction of a more robust model. Calculating the impact of domains on each other and using other evaluation methods could be future goals for the current study.
